# Prevalence of Dry Eye Disease and Its Association With the Frequent Usage of Eye Cosmetics Among Women

**DOI:** 10.7759/cureus.27142

**Published:** 2022-07-22

**Authors:** Norah A Albdaya, Faris H Binyousef, Maha H Alrashid, Abdullah A Alajlan, Faisal A Alsharif, Sulaiman K Alfouzan, Reem R Alhuthail

**Affiliations:** 1 College of Medicine, Imam Mohammad Ibn Saud Islamic University, Riyadh, SAU; 2 Department of Ophthalmology, Imam Mohammad Ibn Saud Islamic University, Riyadh, SAU

**Keywords:** cornea, dry eye syndrome, eyeshadow, mascara, dry eye disease

## Abstract

Purpose: Dry eye disease (DED) is defined as a disease of the tear film and ocular surface that leads to discomfort and visual disturbance. The diagnosis of DED mainly depends on the presenting clinical features. A delay in treatment may progress into chronic eye disease. This study aimed to estimate the prevalence of dry eye symptoms among eye cosmetic users in the Kingdom of Saudi Arabia.

Methods: This cross-sectional study involved adult Saudi females using eye cosmetics. The Ocular Surface Disease Index questionnaire was used to assess DED.

Results: A total of 207 responses were included in this study. DED symptoms were reported among those who used eye cosmetics suggesting their effect on the tear film and its stability. This study demonstrated that mascara is the most common cosmetic used, with a prevalence of 98.6%. The prevalence of dry eye syndrome (DES) was 71.6%, where 40.5%, 13.5%, and 17.6% had severe, moderate, and mild conditions, respectively. The frequency of using inner eyeliner significantly increased the prevalence of DES, in which those who used it daily accounted for 75%. In contrast, the results showed no correlation between the frequency of using mascara or external eyeliner and the prevalence of DES.

Conclusion: The prevalence of DED among women who used eye cosmetics was much higher than its prevalence in the general population, which indicates that eye cosmetics are one of the risk factors in the development of DED. Also, the severity of DED was significantly higher in women who did not use a cleanser for removing cosmetics.

## Introduction

Dry eye disease (DED) is a common condition seen regularly in ophthalmology clinics and is associated with multiple factors [1]. It is a disease of the tear film and ocular surface that leads to discomfort, visual disturbance and tear film variability that can harm the ocular surface. In addition, it is associated with an elevated osmolarity of the tears and inflammation of the ocular surface [1]. The diagnosis of DED mainly depends on the presenting clinical features [2]. A study done in Al-Ahsa, Saudi Arabia, showed the prevalence of dry eye symptoms to be 32.1% [3]. Another study conducted in Riyadh City, Saudi Arabia, estimated the prevalence of DED among adults aged 40 and above to be 34%. The prevalence was higher among females, similar to the Al-Ahsa study [4]. The prevalence of DED increased with age, and there was a significant gender difference as females had a higher incidence of getting DED than males [5,6]. Likewise, Asian ethnicity, old age, smoking, and wearing contact lens were associated with a significantly higher likelihood for dry eye symptoms in a study, as participants frequently reported worse symptoms [3,7].

There was also an association found between dry eye syndrome (DES) and systemic diseases such as arthritis, diabetes, thyroid disease, and others [8]. Moreover, a recent study revealed that dry eye symptoms were reported among those who used eye cosmetics [9]. This suggests that eye cosmetics can change the tear film and its stability [6]. However, there is a lack of studies that support the link between the usage of cosmetics and change in the tear film [10]. Eye cosmetics are used daily in many parts of the world [10]. Eye cosmetics are considered important by both men and women, to increase facial attractiveness [9]. Although the safety of eye cosmetics is guaranteed, through testing, before humans use them, many report an increase in ocular discomfort [10]. Side effects of eye cosmetics range from simple irritation to corneal epithelium inflammation and dry eye [11]. Although many studies have addressed the effect of cosmetics on dry eye symptoms, the effect of different types of cosmetics and the frequency of their usage has not yet been established [10]. If dry eye symptoms are left untreated, they may turn into a progressive and chronic disease and affect the quality of life [6]. However, to our knowledge, there is relatively little published literature on the long-term side effects of eye cosmetic use; hence, this study aimed to evaluate the prevalence and severity of DED among eye cosmetic users, and to explore any relationship between ocular fatigue and cosmetic usage in Riyadh, Kingdom of Saudi Arabia.

## Materials and methods

Study design and population

This randomized, cross-sectional study was performed in Riyadh, Saudi Arabia. The study population included adult Saudi females who used cosmetics or had DED. Participants under the age of 18, who did not sign the informed consent, males, or those with comorbidities such as rheumatoid arthritis, seborrheic dermatitis, Sjogren syndrome, diabetes mellitus, spring catarrh, or glaucoma were excluded.

Study tool and method

The questionnaire had three sections: demographic data, questions related to eye cosmetics usage, and lastly, a well-developed tool to assess DED. The Ocular Surface Disease Index (OSDI) questionnaire was used to assess the symptoms and severity [12]. It is a 12-item validated questionnaire that measures DED severity on a scale of 0 to 100 and can distinguish between normal subjects and patients with DES. The higher the score, the greater the disability. The Arabic version of this questionnaire was validated by a pilot study including 20 random individuals to assure comprehension and ease of administration and identify any issues with the content and language. After obtaining the Institutional Review Board (IRB) approval from Imam Mohammad Ibn Saud Islamic University's Research Ethical Committee, the questionnaire was distributed electronically through an online platform and the study objectives were clarified to the participants. We ensured that no personal information would be taken, and all the participants provided their consent before answering the questionnaire. We performed all statistical analyses using SPSS, version 21 (IBM Corp., Armonk, NY), and a p-value lower than 0.05 was considered statistically significant for all analyses.

## Results

In this study, we were able to collect 320 responses. However, we excluded 113 responses for the following reasons: 65 (20.31%) participants indicated that they had other comorbidities as systemic diseases, including diabetic mellitus, thyroid disorders, rheumatoid arthritis, seborrheic dermatitis, and eye conditions including spring catarrh and glaucoma, while 20 (6.25%) responses indicated that they did an eye surgery within the last year; 18 (5.63%) participants reported using some medications that could cause dry eye. All the previous reasons were excluded because it could cause some conflict and interrupt the study results. Finally, 10 (3.1%) were excluded because they did not complete the questionnaire or were non-Saudi. Therefore, the net sample was composed of 207 (64.6%) responses. Age was categorized into three different age groups: 18-30, 31-40, and above 40 years; 87.9% were in the age group of 18-30 years. Moreover, 75.8% of participants indicated that they had university-level education, and 68.6% were students at the time of the study. Regarding eye problems, 76.8% stated that they had no eye problem, while about a quarter of the participants reported eye dryness. Furthermore, we found that 58.5% of participants indicated using four or more cosmetics for eyes and 17.9% were using moisturizing eye drops (Table 1).

**Table 1:** Demographics of study participants

		N	%
Age (years)	18-30	182	87.9%
	31-40	19	9.2%
	>40	6	2.9%
Education level	Secondary school	42	20.3%
	University	157	75.8%
	Higher education	8	3.9%
Profession	Student	142	68.6%
	Employee	27	13.0%
	Retired	5	2.4%
	Housekeeper	27	13.0%
	Other	6	2.9%
Eye problem	Eye dryness	48	23.2%
	None	159	76.8%
Number of cosmetics most often used	None	0	0.0%
	One	13	6.3%
	Two	24	11.6%
	Three	49	23.7%
	Four or more	121	58.5%
Used medication	Moisturizing eye drops	37	17.9%
	Nothing	170	82.1%

According to participants, mascara and eye shadow were the most commonly used eye cosmetics, with a prevalence of 98.6% and 91.8%, respectively, followed by external eyeliner (78.8%). In comparison, the least used eye cosmetics include false eyelashes (35.3%), inner eyeliner (47.3%), and lenses (54.1%). Mascara was the most commonly used eye cosmetic when it came to daily use, with 18.8% of women using it daily, followed by external eyeliner (7.7%) and eye shadow (5.3%) (Table 2).

**Table 2:** The frequency of using eye cosmetics

	Do not use them, N (%)	Less than once a month, N (%)	Once a month, N (%)	Once or twice a week, N (%)	3-4 times a week, N (%)	Daily, N (%)
How often do you use inner eyeliner?	109 (52.7%)	20 (9.7%)	37 (17.9%)	21 (10.1%)	12 (5.8%)	8 (3.9%)
How often do you use external eyeliner?	46 (22.2%)	55 (26.6%)	45 (21.7%)	26 (12.6%)	19 (9.2%)	16 (7.7%)
How often do you use false eyelashes?	134 (64.7%)	36 (17.4%)	21 (10.1%)	8 (3.9%)	5 (2.4%)	3 (1.4%)
How often do you use eye shadow?	17 (8.2%)	38 (18.4%)	68 (32.9%)	60 (29.0%)	13 (6.3%)	11 (5.3%)
How often do you use the lenses?	95 (45.9%)	46 (22.2%)	28 (13.5%)	12 (5.8%)	17 (8.2%)	9 (4.3%)
How often do you use mascara?	3 (1.4%)	24 (11.6%)	34 (16.4%)	72 (34.8%)	35 (16.9%)	39 (18.8%)

Also, we found that 33.3% of participants indicated the use of cosmetic lenses while 15.9% wore them for medical purposes and 37.7% wore them for more than one year. Furthermore, 34.3% participants indicated that they always used a cleanser to remove cosmetics, while 24.6% said that they rarely used it. Most of them would remove any cosmetic before sleeping (82.6%) and 15.9% participants would sleep with their eye cosmetics (Table 3).

**Table 3:** Participants' routine care, including the type of lenses and use of cleanser

		N	%
Type of lenses used	Not applicable	105	50.7%
	Medical lenses	33	15.9%
	Cosmetic lenses	69	33.3%
Duration since the start of using cosmetics	Not applicable	118	57.0%
	<6 months	11	5.3%
	6-12 months	0	0.0%
	>12 months	78	37.7%
Use of a cleanser to remove cosmetics	Not applicable	9	4.3%
	Rarely	51	24.6%
	Sometimes	76	36.7%
	Always	71	34.3%
Sleep routine	Sleep with eye cosmetics	33	15.9%
	Sleep with lenses	2	1.0%
	Sleep with both lenses and cosmetics	1	0.5%
	Remove lenses or any cosmetic before sleeping	171	82.6%

**Figure 1:**
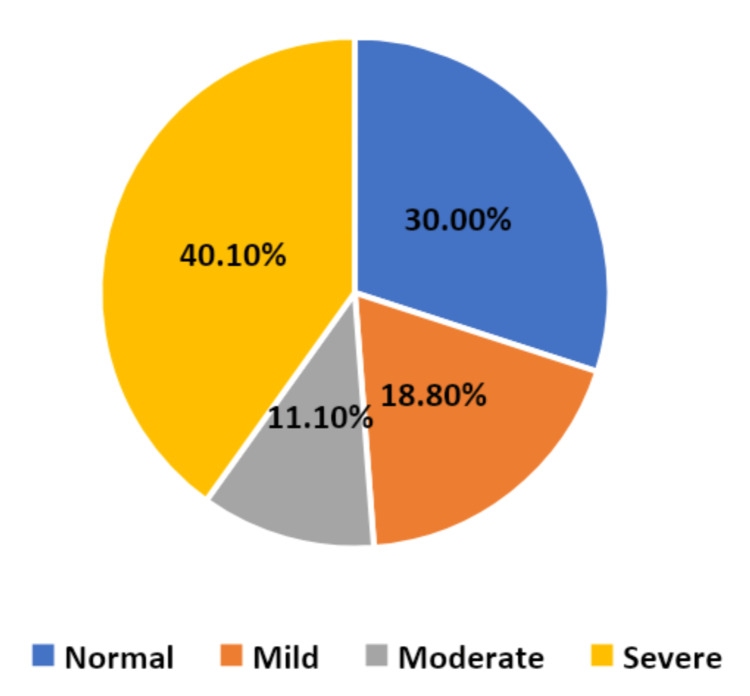
Prevalence of DES according to the OSDI tool and severity of conditions DES, dry eye syndrome; OSDI, Ocular Surface Disease Index

According to the OSDI tool applied in this study, we found that DES prevalence among women who used eye cosmetics was 70%, where 40.1% women had severe conditions, 18.8% had mild, and 11.1% had moderate conditions of DES (Figure 1).

In Table 4, we show the relation between demographic factors of participants and prevalence of DES among the participants; we found that the prevalence and severity of DES were significantly higher in older participants than younger ones (p=0.019). Our results also indicated that the prevalence of DES was statistically significant in the retired population and those employed (p=0.002). Considering the number of used cosmetics and their effect on developing DES, we found no significant difference in the prevalence and severity among participants in whom number of eye cosmetics used varied. In contrast, we found a significant difference in the severity of dry eye conditions in women when the frequency of removing makeup using a cleanser (p=0.018) was considered. The seriousness of DED was higher in women who did not use a cleanser for removing cosmetics. Moreover, we found that the prevalence and severity of DED were significantly higher in women who slept with eye cosmetics than those who removed them before sleeping (p=0.002). Finally, we found that the prevalence and severity of DED were significantly higher in women who did not use moisturizing eye drops (p=0.003).

**Table 4:** The relation between demographic factors and dry eye disease *Significant at p<0.05

		Eye dryness				p-value
		Normal	Mild	Moderate	Severe	
		N (%)	N (%)	N (%)	N (%)	
Age (years)	18-30	60 (33.0%)	37 (20.3%)	22 (12.1%)	63 (34.6%)	0.019*
	31-40	2 (10.5%)	1 (5.3%)	1 (5.3%)	15 (78.9%)	
	>40	0 (0.0%)	1 (16.7%)	0 (0.0%)	5 (83.3%)	
Education level	Secondary school	10 (23.8%)	5 (11.9%)	6 (14.3%)	21 (50.0%)	0.537
	University	51 (32.5%)	32 (20.4%)	16 (10.2%)	58 (36.9%)	
	Higher education	1 (12.5%)	2 (25.0%)	1 (12.5%)	4 (50.0%)	
Profession	Student	41 (28.9%)	32 (22.5%)	15 (10.6%)	54 (38.0%)	0.002*
	Employee	7 (25.9%)	3 (11.1%)	8 (29.6%)	9 (33.3%)	
	Retired	0 (0.0%)	1 (20.0%)	0 (0.0%)	4 (80.0%)	
	Housekeeper	9 (33.3%)	3 (11.1%)	0 (0.0%)	15 (55.6%)	
	Other	5 (83.3%)	0 (0.0%)	0 (0.0%)	1 (16.7%)	
Number of cosmetics most often used	None	0 (0.0%)	0 (0.0%)	0 (0.0%)	0 (0.0%)	0.288
	One	2 (15.4%)	2 (15.4%)	2 (15.4%)	7 (53.8%)	
	Two	7 (29.2%)	3 (12.5%)	3 (12.5%)	11 (45.8%)	
	Three	18 (36.7%)	14 (28.6%)	5 (10.2%)	12 (24.5%)	
	Four or more	35 (28.9%)	20 (16.5%)	13 (10.7%)	53 (43.8%)	
Use of a cleanser to remove cosmetics	Do not use them	1 (11.1%)	2 (22.2%)	0 (0.0%)	6 (66.7%)	0.018*
	Rarely	23 (45.1%)	12 (23.5%)	2 (3.9%)	14 (27.5%)	
	Sometimes	23 (30.3%)	8 (10.5%)	11 (14.5%)	34 (44.7%)	
	Always	15 (21.1%)	17 (23.9%)	10 (14.1%)	29 (40.8%)	
Sleep routine	Sleep with eye cosmetic	11 (33.3%)	5 (15.2%)	2 (6.1%)	15 (45.5%)	0.002*
	Sleep with lenses	0 (0.0%)	0 (0.0%)	2 (100.0%)	0 (0.0%)	
	Sleep with both lenses and cosmetic	0 (0.0%)	0 (0.0%)	1 (100.0%)	0 (0.0%)	
	Sleep without them	51 (29.8%)	34 (19.9%)	18 (10.5%)	68 (39.8%)	
Use of moisturizing eye drops	No	60 (34.9%)	32 (18.6%)	19 (11.0%)	61 (35.5%)	0.003*
	Yes	2 (5.7%)	7 (20.0%)	4 (11.4%)	22 (62.9%)	

Increasing the frequency of using an inner eyeliner significantly increased the prevalence of DES from 55% in those using it less than once a month to 75% in those who used it daily (p=0.001). Furthermore, we found that those who used false eyelashes, eye shadow, and lenses on a daily basis showed the highest and the most significant impact on their eyes, where all participants who used these cosmetics daily had DES. Its prevalence increased significantly with increasing the frequency of using them. However, no significant difference was found in the majority of DES cases when the frequency of using mascara or an external eyeliner (p=0.234, p=0.058, respectively) was considered (Table 5).

**Table 5:** The relation between the type and frequency of using eye cosmetics and dry eye disease *Significant at p<0.05

		Eye dryness				p-value
		Normal	Mild	Moderate	Severe	
		N (%)	N (%)	N (%)	N (%)	
Inner eyeliner use	Do not use	37 (33.9%)	31 (28.4%)	12 (11.0%)	29 (26.6%)	0.001*
	Less than once a month	9 (45.0%)	1 (5.0%)	3 (15.0%)	7 (35.0%)	
	Once a month	8 (21.6%)	2 (5.4%)	2 (5.4%)	25 (67.6%)	
	Once or twice a week	4 (19.0%)	3 (14.3%)	4 (19.0%)	10 (47.6%)	
	3-4 times a week	2 (16.7%)	2 (16.7%)	0 (0.0%)	8 (66.7%)	
	Daily	2 (25.0%)	0 (0.0%)	2 (25.0%)	4 (50.0%)	
External eyeliner use	Do not use	16 (34.8%)	15 (32.6%)	4 (8.7%)	11 (23.9%)	0.058
	Less than once a month	19 (34.5%)	10 (18.2%)	9 (16.4%)	17 (30.9%)	
	Once a month	12 (26.7%)	4 (8.9%)	5 (11.1%)	24 (53.3%)	
	Once or twice a week	9 (34.6%)	4 (15.4%)	3 (11.5%)	10 (38.5%)	
	3-4 times a week	2 (10.5%)	5 (26.3%)	1 (5.3%)	11 (57.9%)	
	Daily	4 (25.0%)	1 (6.3%)	1 (6.3%)	10 (62.5%)	
False eyelash use	Do not use	48 (35.8%)	28 (20.9%)	16 (11.9%)	42 (31.3%)	0.014*
	Less than once a month	6 (16.7%)	7 (19.4%)	4 (11.1%)	19 (52.8%)	
	Once a month	8 (38.1%)	2 (9.5%)	2 (9.5%)	9 (42.9%)	
	Once or twice a week	0 (0.0%)	0 (0.0%)	1 (12.5%)	7 (87.5%)	
	3-4 times a week	0 (0.0%)	2 (40.0%)	0 (0.0%)	3 (60.0%)	
	Daily	0 (0.0%)	0 (0.0%)	0 (0.0%)	3 (100.0%)	
Eye shadow use	Do not use	7 (41.2%)	4 (23.5%)	0 (0.0%)	6 (35.3%)	0.09
	Less than once a month	12 (31.6%)	11 (28.9%)	2 (5.3%)	13 (34.2%)	
	Once a month	24 (35.3%)	11 (16.2%)	8 (11.8%)	25 (36.8%)	
	Once or twice a week	18 (30.0%)	9 (15.0%)	10 (16.7%)	23 (38.3%)	
	3-4 times a week	1 (7.7%)	4 (30.8%)	1 (7.7%)	7 (53.8%)	
	Daily	0 (0.0%)	0 (0.0%)	2 (18.2%)	9 (81.8%)	
Lenses' use	Do not use	44 (46.3%)	17 (17.9%)	5 (5.3%)	29 (30.5%)	0.00*
	Less than once a month	8 (17.4%)	14 (30.4%)	4 (8.7%)	20 (43.5%)	
	Once a month	6 (21.4%)	4 (14.3%)	7 (25.0%)	11 (39.3%)	
	Once or twice a week	2 (16.7%)	3 (25.0%)	3 (25.0%)	4 (33.3%)	
	3-4 times a week	2 (11.8%)	1 (5.9%)	2 (11.8%)	12 (70.6%)	
	Daily	0 (0.0%)	0 (0.0%)	2 (22.2%)	7 (77.8%)	
Mascara use	Do not use	0 (0.0%)	1 (33.3%)	0 (0.0%)	2 (66.7%)	0.234
	Less than once a month	9 (37.5%)	4 (16.7%)	1 (4.2%)	10 (41.7%)	
	Once a month	11 (32.4%)	8 (23.5%)	3 (8.8%)	12 (35.3%)	
	Once or twice a week	26 (36.1%)	10 (13.9%)	9 (12.5%)	27 (37.5%)	
	3-4 times a week	10 (28.6%)	11 (31.4%)	5 (14.3%)	9 (25.7%)	
	Daily	6 (15.4%)	5 (12.8%)	5 (12.8%)	23 (59.0%)	

**Figure 2:**
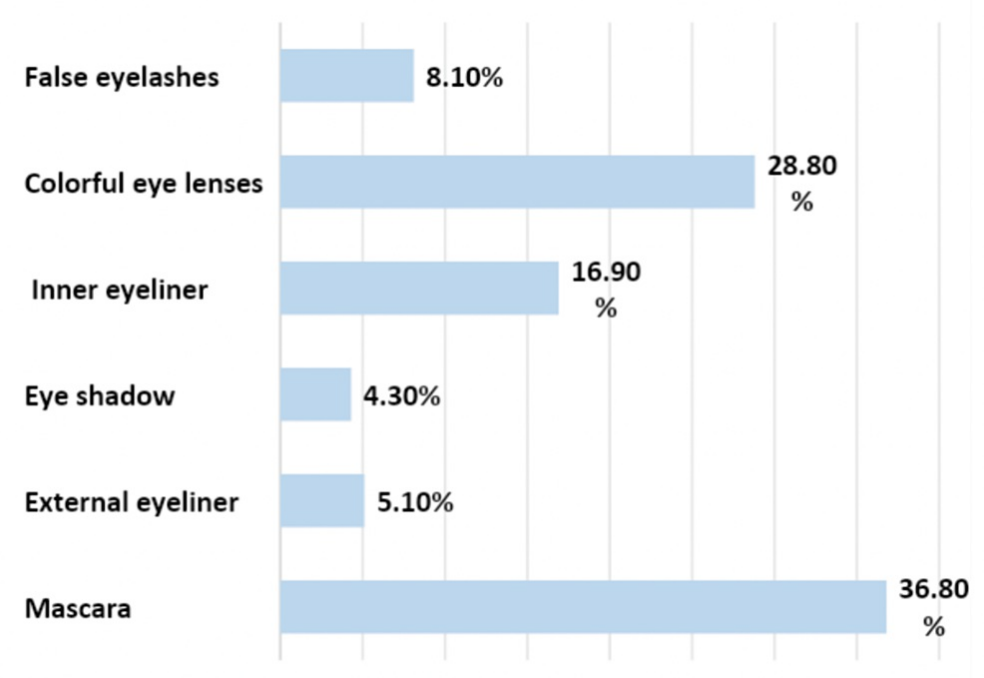
Cosmetics with the most negative impact on eye dryness according to participants

Regarding participants’ perception, majority of them agreed that mascara was the main cosmetic that could lead to the incidence of DES (36.8%) followed by colorful eye lenses (28.8%) and inner eyeliner (16.9%) (Figure 2).

## Discussion

Cosmetics are a symbol of femininity, representing stereotypical feminine values. When body care products, particularly cosmetic products, are absorbed into the skin, they penetrate and reach the underlying tissues. According to the latest statistics, cosmetics are widely used by Saudi students and women. Studies show that Saudi women wear more makeup than women from the Arab world, the West, and the Persian Gulf. It was found that the number of imported cosmetics exceeded 2.3 billion in 2015 [13]. Our study discovered that about 60% of participants would use more than four types of cosmetics, with mascara, eye shadow, and external eyeliner being the most popular. A study conducted by Shaaban and Alhajri among the female population of Saudi Arabia revealed that mascara, eyeliner, eyebrow pencil, and eye shadow were the most commonly used eye cosmetics among this population [14].

Considering the usage of cosmetic eye lenses, we found that half of the sample indicated using lenses and 33% of the participants used them for cosmetic purposes. A study conducted in Saudi Arabia found the prevalence of contact lens usage among Saudi Arabians to be 15% [15]. Moreover, we found that 24.6% of participants rarely used eye cosmetic cleansers; 4.3% never used them at all and those showed statistically significant severity of DED. Our results were broadly in line with another study that stated that using an eye cleanser can maintain the meibomian gland and prevent dry eye symptoms [16]. Previous studies have consistently reported the migration of cosmetic products to the eye surface, and tear film contamination from cosmetic products has been observed [11,17-19]. The studies established that the accidental migration of cosmetic products to the periocular area could provoke tear film contamination [17]. This phenomenon is assumed to be involved in some reported ophthalmic side effects, including posterior blepharitis, eye irritation, tear film instability, conjunctival pigmentation, corneal epithelium defect, and keratitis [20-22].

In our study, the prevalence of DED among women who used eye cosmetics was 71.6%, where 40.5% of women had severe conditions, 17.6% had mild, and 13.5% had moderate conditions of DED. This prevalence among the eye cosmetic user population is much higher than reported among other populations, including old hospital patients in which the prevalence of DED was 54.3% [23]. However, it is known that the prevalence of DED is higher in aging populations than younger ones [24]. This was supported by our study as DED was significantly higher in older participants. Moreover, the prevalence of DED among the normal population ranged from 7.4% to 33.7%, which is much lower than the prevalence detected in our population [25,26]. As mentioned earlier, in the Al-Ahsa region, the prevalence of DED was estimated to be 32.1% among the normal population [3]. The higher prevalence in our population indicates that there is a relationship between the usage of eye cosmetics and a high prevalence of DED; the difference in prevalence may be due to different tools used in the diagnosis of DED despite it still being a good indicator toward the relationship between eye cosmetics and their negative impact on the eye health. In addition, many previous studies have verified that women tend to suffer from DED more than men, which may further correlate eye cosmetics and dry eye symptoms, as women tend to use cosmetics more frequently [3,24,25,27].

However, we found that the prevalence of DED increased significantly with an increase in the frequency of using some eye cosmetics applied to periocular skin, including inner eyeliners, false eyelashes, eye shadows, and eye lenses. This result indicates that the negative impact of eye cosmetics on the eye increases based on two conditions: the application of cosmetics near the eye and the high frequency of usage of these cosmetics. A similar pattern of results was obtained in another study that found that eye cosmetic wearers displayed marginally poorer OSDI scores than participants who did not use eye cosmetics. However, the study did not find a correlation between the usage frequency and specific product applied and the prevalence of DED [9]. Another study demonstrated that applying eyeliner for one week negatively contributed to ocular comfort [28].

This study included some unavoidable limitations. First, using a self-reported questionnaire may lead to some personal bias where some participants may not answer all questions honestly to appear in a better form, reducing the percentage of their use of cosmetics on purpose. Besides, the sample size was relatively smaller than expected, which is believed to be due to an increase in online surveys. As a result, people tend to become less motivated toward online surveys. Concurrently, to follow preventive measures, an online survey was the best solution to conduct our study.

## Conclusions

In conclusion, we found that the prevalence of DED among women who used eye cosmetics was 71.6%, which is much higher than the prevalence of DED in other populations, indicating that using eye cosmetics is a risk factor for developing DED. We believe that the negative impact of eye cosmetics on eye health increases based on two conditions: the application of cosmetics near the eye and the high frequency of usage of these cosmetics. Further studies with objective assessments of DED with a more reliable diagnostic approach are needed to further assess the effect of cosmetics on DED.
